# Global Medicine, Parasites, and Tasmania

**DOI:** 10.3390/tropicalmed5010007

**Published:** 2020-01-01

**Authors:** John Goldsmid, Silvana Bettiol

**Affiliations:** School of Medicine, College of Health and Medicine, University of Tasmania, 17 Liverpool Street, Hobart Tasmania 7000, Australia; j.m.goldsmid@utas.edu.au

**Keywords:** parasitology, zoonoses, tropical medicine, travel medicine, global medicine, Tasmania

## Abstract

Until the 1970s, infectious disease training in most medical schools was limited to those diseases common in the area of instruction. Those wishing to explore a more globalised curriculum were encouraged to undertake specialist postgraduate training at schools or institutes of tropical medicine. However, the increase in global trade and travel from the 1970s onward led to dramatic changes in the likelihood of returning travellers and new immigrants presenting with tropical infections in temperate regions. Furthermore, population growth and the changing relationships between animals, the environment, and man in agriculture accentuated the importance of a wider understanding of emerging infectious diseases, zoonotic diseases and parasitic infections. These epidemiological facts were not adequately reflected in the medical literature or medical curriculum at the time. The orientation on tropical infections needed specialised attention, including instruction on diagnosis and treatment of such infections. We describe key global health events and how the changing field of global medicine, from the 1970s to early 2000, impacted on medical education and research. We describe the impact of global health changes in the Tasmanian context, a temperate island state of Australia. We retrospectively analysed data of patients diagnosed with parasites and present a list of endemic and non-endemic parasites reported during this period. Finally, we reflect on the new approaches to the changing needs of global health and challenges that medical programmes, learners and educators face today.

## 1. Introduction

In 1964 and based on concerns regarding tropical disease in temperate climates, Dr. Kevin Cahill published a series of articles in The New York State Journal of Medicine. He foresaw that inexpensive boat travel and the shortening transit times by air travel posed a potential infection hazard [1]. By 1974, Woodruff in the preface to his book stated that “Medicine in the tropics is of great importance to all practitioners be they working in temperate or tropical regions” [2]. He had already noted that through travel “even in temperate regions, a significant proportion of the community has now been exposed to disease in tropical and subtropical regions” [2]. What he said then is even more important now with more global connections involving travel and trade, changes in climate, and the environment and human spread into previously uninhabited regions. These factors have led to the possibility of diseases with short incubation periods being brought to countries where they have not been seen before or have been exceedingly rare. Diseases with long and silent incubation periods present the most difficult diagnostic and public health problems. This, has led to growing clinical needs and development of medical practitioners, public health and health professionals with an understanding of these diseases, working at the local, regional, national, and global level. For those teaching and developing medical curricula these diseases present many challenges and have become more complex due to the changing focus of healthcare systems, globalisation, cultural and societal factors, and technology. Despite international communications on medical education to address these needs, medical practice remains distinctly different among countries [3].

## 2. Time Line of Selected Global Health Events

Why were the 1970s significant for changes in global medicine? The 1970s was a period when the post-World War II economic expansion and economic boom was drawing to an end and the 1973–1975 recession loomed [4,5]. The economic crisis also affected the approach to the control of disease and much of the work in the field reflected the long-term instability and economic difficulty. The range of effective drugs used for human and animal treatment expanded rapidly. However, the treatment was not always in parallel with investigations of how these new drugs should be used to the best effect. Transport of livestock from country to country without an understanding of disease risks and intensive methods of animal management posed hazards to humans [2,6]. Preparing for reduced funding for medical and veterinary services to make further gains in health and wellbeing of humans and animals was not expected. Promoting efficient use of resources was common and a need for evaluation was obvious.

The growing efficiency and reach of modern transport networks led to an emergence of new strains of familiar diseases, as well as completely new diseases. A consequence was the pressure on how to tackle them. It became apparent that there was strong correlation between antibiotic use in the treatment of humans and animals and antibiotic resistance in Gram-negative pathogens. The most prevalent Gram-negative pathogens at this time were *Escherichia coli*, *Salmonella enterica*, and *Klebsiella pneumoniae* [7]. Since then there has been an alarming increase in ‘superbugs’ and a decline in the development of new antibiotics to cope with the changing situation. Williamson et al. [8] recently highlighted the danger of drug resistance in *Candida auris,* carbapenemase-producing enterobacteriaceae, Methicillin-resistant *Staphylococcus aureus* (MRSA) and drug-resistant strains of typhoid and gonorrhoea. Infectious disease specialists note that the world has reached a crisis in the treatment of bacterial infections [9,10].

Between 1965 and 1970, the growth rate of the world’s population reached its peak, increasing by 2.1% per year on average [11]. There was a growing concern of the impact of this population growth on the interaction of humans and the environment and how this would affect tropical diseases. A combination of increased economic activity, human migration, tourism, and encroachment on new environmental niches contributed to the emergence of many zoonotic diseases. The first cases of naturally acquired *Plasmodium knowlesi* infection [12], the Marburg, Ebola, and Lassa fever viruses [13], and reports of Lyme disease [14] occurred during this period. The earliest reports of a syndrome later identified as HIV/AIDS appeared in the closing year of the 1970s [14].

The 1970s saw the rise of preventive medicine and the self-care movements [15]. In the early part of June 1972, the United Nations Conference on the Human Environment in Stockholm considered the need for “a common outlook and for common principles to inspire and guide the peoples of the world in the preservation and enhancement of the human environment” [16]. Following the Stockholm Declaration, global awareness of environmental issues increased dramatically. In 1992, the second United Nations Conference on Environment and Development (UNCED) in Rio de Janeiro represented a major milestone in the evolution of international environmental law [17]. Today, the Sustainable Goals (SDGs) of the United Nations and the Agenda 2030 reflect the spirit of these principles.

The 1970s initiated great strides in global disease control. A successful example was the Onchocerciasis Control programme, which commenced in 1974 [18]. The serious health and socioeconomic repercussions of onchocerciasis and indications of possible control was a catalyst for a convening of a joint USAID/OCCGE/WHO technical meeting [18,19]. The participants included experts in a range of fields including public health, parasitology, epidemiology, entomology, ophthalmology, economics, sociology, and medical geography [18]. The programme brought relief to many communities and with great effort was sustained and continues today, despite difficult circumstances. This programme has become an example of effective public health management and one of the largest intercountry undertakings implemented by the World Health Organization (WHO) [19,20].

The decade of the 1970s closed with the successful eradication of smallpox after a 14-year intensive programme [21] and the beginning of new global control programmes. There were specific efforts to increase efficiency and productivity of healthcare systems during the 1980s, including improvement in maternal and child health and a focus on HIV/AIDS, tuberculosis, and malaria in developing countries. In 1986, the Global Programme on AIDS (GPA) was launched by the World Health Organization [22] followed by the Global Polio Eradication Initiative (GPEI) in 1988, which was led jointly by national governments, the WHO, Rotary International, the US Centers for Disease Control and Prevention (CDC), and UNICEF [22,23].

In 1992, the CDC launched the international campaign to eradicate Guinea worm disease and eliminate dracunculiasis [24]. The 1990s welcomed the evolution of large data-driven research and collaboration with the World Bank commissioned to publish the Global Burden of Disease study. Today this collaboration has over 1800 researchers and contributors from 127 countries [25]. Another significant global achievement at this time was the Global Initiative for Traditional Systems of Health created by the Pan American Health Organisation [26]. Today WHO continues to develop proactive policies and action plans to strengthen the role of traditional and complementary medicine (T&CM) in responding to health needs of populations. Unfortunately, it remains an ongoing challenge for countries trying to implement regulations and national laws for their use [26].

The next factor which influenced human health over these years, and more evident today, was climate change. Changing climate, spread of warmer conditions, and changing rainfall can increase the occurrence of tropical diseases such as malaria, dengue and schistosomiasis and potentially soil transmitted helminthiases by extending their distribution [27]. Changing climate, including pollution, exacerbates public health issues and economic stagnation due to parasitic diseases and these complexities have been highlighted by numerous authors [28,29,30,31]. Thus, Han et al. [32] stated that “Health organisations are growing more concerned that climate change will cause zoonotic diseases to become more prolific and widespread” and challenges are more difficult in predicting outbreaks caused by either novel pathogens or known pathogens in novel places.

The continued pressure of economic development has increased the opportunity for pathogens (many previously unknown) with zoonotic potential to cross the species line. The world’s population has continued to grow from 3.7 billion in the 1970s to 7.7 billion in 2019 [33] and is projected to increase to 9.7 billion by 2050. Health professionals need to be aware of growth rates and mobility patterns across their regions [34]. Goldsmid [35] noted “that never before in the history of the human race, have so many people been able to travel so far so quickly and so cheaply”—and this is even truer today!

A review of the literature estimates the number of people that acquire an infectious disease during or as a result of travel ranges from 6% to 87% [36]. These figures may change as current projections suggest the annual number of international travellers will reach 1.8 billion by 2030 [36]. International tourism in Australia for example has grown significantly in the past two decades. The number of short-term overseas visitor arrivals rose from 2.5 million in 1992 to 9.3 million in 2018–2019, the highest year on record [37]. Outbound international trips have nearly doubled in the past decade. The scale and speed of contemporary international travel means an increasing possibility of travellers being exposed to unfamiliar infections.

Accurate data of the proportion of people who acquire an illness overseas are difficult to calculate as exposure is dependent on the destination, baseline medical history, and also planned activities [38]. In recent years research in travel medicine has grown. There are tropical medicine surveillance networks, such as the GeoSentinel surveillance network composed of International Society of Travel Medicine (ISTM) travel and tropical medicine clinics that collect post-travel illness surveillance data [39,40], but there are limitations. Therefore, physicians need to be familiar with destination-specific disease risks, travel and routine vaccines, and chemoprophylaxis regimens. Reviews of current evidence in the discipline are readily available [41].

A major area of medical relevance in the fields of tropical travel, migrant and refugee medicine is parasitic infections. The estimates of the prevalence and incidence of neglected tropical diseases and malaria from the Global Burden of Disease Study 2017 [42] are thought provoking. While some parasitic diseases such as falciparum malaria and human African trypanosomiasis (East African variety) cause acute infections with a high mortality, most parasitic infections are chronic infections. An example is cysticercosis [43,44,45] which is increasingly recorded in non-endemic regions around the world [43].

## 3. Historical Overview of Tasmania and Parasites of Medical Importance

Historically, nearly all of the infectious diseases seen in Australia, and especially Tasmania, have been imported since European/Asian settlement of the continent [46] and many of the commoner infections then became endemic as the population grew. Tasmania has a long history associated with imported infections, starting with the first settlement by Europeans (especially the convicts) and continuing from there. Tasmania provides a strong case against a parochial approach to medicine – especially in the changing world today. It is a good model for study in relation to imported infections and the relevance of travel in this regard. The reasons are:

(1) Tasmania is a small relatively isolated island, protected by surrounding water.
(2) Tasmania has a temperate climate with a high standard of living and with good health services.
(3) Tasmania has a small resident population and consequently has fewer overseas travellers or returning travellers.
(4) Tasmania has few direct overseas connections and fewer overseas visitors than the more populous and easily accessible mainland states of Australia.

The question thus arises—How big a range of endemic and ‘exotic’ imported infections has been diagnosed in humans in Tasmania over recent years? A retrospective analysis of recorded cases was completed and is reported below. Helminth infections are summarised in Table 1 and other key parasites diagnosed in Tasmania in Table 2.

In the mid-20th Century, Tasmania experienced one of the highest rates of human hydatid disease in the world [47,48]. *Echinococcus* granulosus, which causes cystic echinococcosis is the only member of the genus *Echinococcus* to be found in Australia. It was introduced during the early period of European settlement and described in domestic animals before 1840 [49]. In the early 1960s, the state government commenced a hydatid control programme across Tasmania aimed at stopping transmission of hydatid disease to humans. By 1996, Tasmania was declared provisionally free of hydatids in dogs and livestock [50].

Of the helminth cases, the Tasmanian cases of *Trichinella pseudospiralis* and *Haycocknema perplexum* are the most intriguing as they were the first human cases of these worms ever reported [51,52,53]. It was first described from a patient in Tasmania by Spratt et al. [54]. Subsequent cases diagnosed had all lived in or visited Tasmania. Many had histories of contact with native animals or eating bush meat and it was thought that the infection was an endemic zoonosis from Tasmania [55,56]. A subsequent case from tropical North Queensland had no contact with Tasmania and a re-evaluation of all the recorded cases showed that they all had contact with the tropical north of Australia. The result that the condition was considered to be endemic there and being redescribed the disease as “tropical parasitic myositis” [57]. However, the most recently reported case from Tasmania had no travel history, except to Melbourne in southern Victoria [56], confirming that *H. perplexum* is indeed endemic to Tasmania. The natural reservoir of *H. perplexum* has not been found to date and the mode of transmission to humans is also unknown. Human *T. pseudospiralis* has subsequently been described from humans in France and Thailand but the enigma of *H. perplexum* continues.

Vaccine development and application to control zoonotic diseases in food animals, companion animals, and wildlife have made a significant impact in reducing the incidence of zoonotic diseases in people [58]. At the same time, biosecurity in Australia has played a critical role in maintaining its reputation as a country free of severe pests and diseases. The Commonwealth Quarantine Service started operations in 1908 and today Biosecurity Australia monitors plant and animal health across Australia. While most infections in Australia have been controlled it has led to over-confidence regarding their importance (or even existence) today. Unfortunately, while ubiquitous protozoan species have been reported in wildlife across Australia [51,52,53,54,55,56,57,58,59,60,61] the life cycles, ecology, and general biology of most parasites of wildlife in Australia are poorly understood. Much of the work to date has been opportunistic with unreliable funding opportunities, but with modern methods and ‘omic’ technology [62] offering an avenue for major advances in the field, there is potential for renewed support and interest.

An analysis of imported malaria in Tasmania (Table 2) was initially reported over a 5-year period from 1987 to 1992 and subsequently extended to cover 1987–1994 [63]. During this period, 80 cases of malaria were diagnosed covering three species: *Plasmodium vivax* (81.2%) followed by *Plasmodium falciparum* (16.2%), and *Plasmodium malariae* (2.6%). Cases came from Asia, Southeast Asia, Africa, Papua New Guinea (PNG), Vanuatu, the Solomons, and one case acquired in Thursday Island (administratively a part of Australia and an area considered free from malaria due to control measures). The cases were diagnosed from across the State, including two from Antarctica—a region administered from Tasmania (Goldsmid, pers. Comm. 2019). These cases continued to influence curriculum development in tropical and travel disease in the undergraduate medical school. Expanding medical training in tropical disease for Tasmanian medical practitioners was highlighted by one of these malaria cases when originally misdiagnosed (as influenza). The general practitioner (GP) involved explained the patient presented during an influenza epidemic in his area and, as he ruefully admitted, he did not ask the patient about any travel history. Luckily it was a case of benign tertian malaria (*Plasmodium vivax*)—the error could have been tragic if it had been a case of *P*. *falciparum* malaria

New treatments are, however, being developed, albeit rather slowly and an example of this is the treatment of *Plasmodium vivax* relapses with tafenoquine (kozenis) and artesunate for severe falciparum malaria [64]. The recent and rapid clinical development of vaccines is making considerable impact world-wide. The vaccine against *Plasmodium falciparum*, RTS,S/AS01 (RTS,S) is a promising vaccine candidate for malaria currently part of a landmark pilot program in three African countries, and is available to children up to 2 years of age [65]. Another recent example is the vesicular stomatitis virus-based Ebola virus vaccine (VSV-EBOV) currently in a clinical trial [66]. It is viewed as a potential tool for both current and future Ebola outbreaks.

Infectious diseases and pandemic preparedness remain a priority on the world agenda, despite the shift in focus to non-communicable diseases. The Sustainable Development Goals (SDGs) provide a framework for a holistic answer to the ensuing challenges and respond to these complex interactions but collaboration between research disciplines is essential. A recent paper by committee members of the World Heath Summit supports the view that leading institutions and organisations are responsible to promote transdisciplinary, cross-sectoral, science-based and concerted efforts for more efficient and equitable ways to advance health on a global scale [67].

## 4. Conclusions

Changing attitudes over the course of several decades mean medicine has slowly been considered in global terms rather than discrete specialties. The changing patterns in disease suggest a need for new conceptual models in medical training and medical education. There have been many attempts to frame the impact of globalisation on health [68] based on international health, public health, and tropical medicine [68,69,70]. During the period of our work in medical curriculum development and medical microbiology teaching the commoner categories included: tropical medicine, travel medicine, migrant medicine, and traditional and complementary medicine. All of these specialties, to varying degrees, can be encompassed under the broad title of ‘Global Medicine’. This has coincided with attempts to integrate modern science into the medical curricula and equip health professionals to meet the future health needs of populations.

The One Health concept recognises the health connections between humans, animals, and their shared environments [71]. Its approach has been endorsed by a number of medical and public health organisations and medical schools around the world [71]. The concept of planetary health, which became part of the fabric of integrative medicine in the 1990s, has since become a concept that has penetrated mainstream academic and medical discourse. In fact, it provides a new multidisciplinary approach to understanding the interconnections between environmental and human health. It is often viewed as a response to existing fields of public health, environmental health, Ecohealth, One Health, and international health [72].

The impressive range of infections currently seen and potentially emergent emphasises what Bradley [73] warned the medical profession over 30 years ago, that a holistic view is one all specialties must embrace. Our work in medical education and our past experiences are still valid today. Tropical diseases are not confined to the tropics. They are being increasingly encountered in non-tropical areas and thus must be considered a global problem.

## Figures and Tables

**Table 1:** Helminth infections diagnosed in Tasmania:

Trematodes:	Cestodes:	Nematodes:
*Echinostoma* sp. **	Echinococcus granulosis ***	Ancylostoma duodenale **
Fasciola hepatica **	Hymenolepis nana ***	Ascaris lumbricoides ***
Opisthorchis viverrni **	Taenia saginata **	Ascaris suum *
Schistosoma haematobium **	*Taenia solium* (cysticercosis) **	Cutaneous larva migrans **
Schistosoma mansoni **		Enterobius vermicularis *
		Eucoleus aerophilus (Capilalria aerophila) *
		Haycocknema perplexum **
		Loa loa **
		Necator americanus **
		Strongyloides stercoralis **
		*Toxocara* spp. *
		Trichinella pseudospiralis *
		Trichuris trichiura ***
		*Trichostrongylus* spp. **
		*Wuchereria bancrofti* lymphatic filariasis and tropical pulmonary eosinophilia **

Key to coding: Endemic cases *, Imported cases **, Endemic and imported cases ***.

**Table 2:** Protozoa and Arthropoda diagnosed in Tasmania.

Protozoa	Arthropoda—Insecta:	Arthropoda—Acarina
Chilomastix mesnili ***	Cordylobia anthropophaga **	Sarcoptes scabiei *
*Cryptosporidium* spp. *	Dermatobia hominis **	Ixodid ticks *
Cyclospora cayetanensis **	Tunga penetrans **	
Dientamoeba fragilis ***	Pediculus humanus capitis *	
Endolimax nana ***	Pediculus humanus corporis *	
Entamoeba histolytica **		
Entamoeba dispar **		
Entamoeba polecki **		
Entamoeba gingivalis *		
Enteromonas hominis **		
Giardia intestinalis ***		
Leishmania tropica **		
Leishmania braziliensis **		
Pentatrichomonas hominis **		
Plasmodium falciparum **		
Plasmodium malariae **		
Plasmodium ovale **		
Plasmodium vivax **		
Toxoplasma gondii *		
Trichomonas vaginalis *		
Trypanosoma cruzi **		
Acanthocephala; Moniliformis moniliformis **		**

Key to coding: Endemic cases *, Imported cases **, Endemic and imported cases ***.
